# Case of Abdominal Inflammation with Some Remarks

**Published:** 1818-07-01

**Authors:** 


### *Case of Abdominal Inflammation with some Remarks.* By a PHYSICIAN in London.*

After exposure to cold, about the end of January last, a gentleman, aged 41, began to complain of chilliness,

---

* The Editors answer for the authenticity of this case, and the high authority of the writer.

with general oppression, loss of appetite, and load at the stomach. Notwithstanding these symptoms remained with little variation, he continued to perform his usual business for upwards of three days, when, for the first time, he was attacked with severe, but only occasional, pain in the bowels, accompanied with the signs of fever. The pain was urgent for several hours together during the next four days, but there were intervals of complete ease, though the fever never wholly abated. At the expiration of the last mentioned period, a respectable practitioner was called in, who prescribed a brisk purgative, which gave considerable relief to all the symptoms, more especially to the pain. As the patient, however, was deemed to be in danger, a physician was consulted on the following morning, and the pain was then so trifling that it could only be indistinctly felt on forcible pressure near the umbilicus, where the integuments, for a limited space, were rather tender to the touch. In contravention to the seeming subsidence of the most urgent symptoms, the pulse was above 130 in the minute, the stomach flatulent, the tongue loaded, the thirst intense, and the voluntary powers more impaired than before. Under an idea that inflammation still probably existed within the cavity of the belly, some leeches were applied near the scite of the uneasiness, and another purgative administered. These measures removed the slight remains of pain, the pulse fell to 110 in the minute, and the patient declared himself better in every respect. An anodyne was prescribed at night, which nevertheless was passed in a restless state, though there was no return of pain; but on the ensuing morning, the pulse rose to 140 in the minute, and the whole system became highly irritable. Some doses of calomel and opium were exhibited in the day, and before bed-time the pulse fell to 106 in the minute; yet as the pain and soreness of the belly returned, the leeching and purging were once more ordered, and with the same relief of symptoms as in the former instance. The patient, however, passed another restless night, and was rather delirious, while the pulse continued to become quicker; the irritation greater, in spite of opiates, and he sunk exhausted the next day. On opening the body, about twenty-four hours after death, the large and small intestines shewed the most striking signs of inflammation, and the right lobe of the liver was found harder and more contracted than natural, from a previously chronic disease of that organ.

This case is remarkable for the stages, peculiar symptoms, and morbid appearances. For about four days, the patient was chilly, languid, and oppressed with many of those marks of venous internal congestion, which a recent author has described as the concomitants of the cold stage; and with the febrile re-action, a pain of the bowels afterwards arose, which had a spasmodic character, since it abated and returned for four days distinctly, and was never of long duration after that time. As the pain finally disappeared, exhaustion and irritation, with vomiting, supervened, under which the patient expired; and dissection fully revealed that an inflammatory action had existed with the fever, though the pulse sometimes fell, and the pain was not permanent. It is clear from the history of this case, that the most precious time was lost before the obtainment of medical advice, for both the cold stage of collapse, and the greater part of that re-action had passed away, so that the practitioners had to encounter the disease at a late period, when the morbid actions were probably too confirmed for removal; or when, at all events, the strength was too much exhausted to admit of more vigorous measures than those which were employed. The chronic affection of the liver had, perhaps, retarded the return of venous blood from the bowels, and hence they had been made so susceptible, that when a general impression from cold occurred, the inflammation was ultimately excited; and in all probability, too, the affection of the liver had tended to aggravate the intestinal inflammation, when it actually took place, and no doubt made it more unmanageable. Yet this example may serve to shew how dangerous it is to neglect obtaining advice in diseases ushered in by a cold stage; because when that stage is allowed to go on uninterruptedly, it is almost sure to lead to inflammation in the end, when any part happens to be predisposed to that condition. The prompt use of the warm bath, and of purgatives, is among the best expedients for removing the symptoms of the cold stage, and blood-letting is sometimes necessary in the more marked examples, after the bath; but where there is pain, whether constant or occasional, in the hot stage, the lancet should always be early used, and followed up quickly by purgatives, with calomel and opium. These means will generally answer the purpose, in the beginning of the fever and pain; yet where these symptoms have run on, so as to produce exhaustion and irritation, the result will commonly be fatal, whatever means be pursued; and this will more particularly happen where an acute inflammation supervenes on a chronic disease, as occured in the case which has just been reported.

*London, March,* 1818.

---

To the above very interesting case, we shall add a short sketch of two others, recently related by Dr. Hurtado, of Madrid, shewing the danger and the treachery of abdominal inflammation.

*Case* 1.—A medical gentleman, residing in a village about two leagues from Madrid, 50 years of age, of a frail constitution and irritable temperament, after some anxiety occasioned by a literary composition, was seized at midnight with what was called a severe spasmodic colic, for which he took a grain of opium, and shortly afterwards the warm bath. The disease, however, increasing in violence, he sent, next morning, for the learned Sevano Lopez, first professor of the clinical school in Madrid, who unfortunately was unable to visit the patient till after mid-day. He found the gentleman perfectly tranquil; but this calm, the professor saw, was the forerunner of death, which took place at midnight, in despite of every medical measure. No dissection was made.

*Case* 2.—Dr. Hurtado was called to a man who had been suddenly seized with colic, the violent pain of which caused the friends to run in all directions for medical assistance, so that another physician had arrived before Dr. H. and had prescribed such medicines as he thought necessary. The patient now expressed himself perfectly relieved, and Dr. H. after learning the history of the complaint, and the remedies exhibited (which consisted of anodynes internally; fomentations to the abdomen; glysters of poppy decoction and olive oil, &c.) considered the patient as entirely free from danger, and approved of the means used by the other physician. He was not a little surprised the next day by an invitation from the physician in attendance, to assist at the dissection of the patient, who expired three hours after Dr. Hurtado left the house! The pain had returned with violence, succeeded by cold sweats, syncope, fœtid evacuations by stool, and death.

*Post mortem appearances.*—On the peritoneal surface of the small intestines many violet spots. The beginning of the colon was contracted, and its parietes thickened. On its interior surface were scattered several small hard granular eminences, like an herpetic eruption. The large intestines also contained a kind of greyish transudation [ espece de transudation grisatre ] of very fœtid odour. All the other viscera were sound.

The three foregoing cases present a striking contrast between the therapeutic, as well as the pathological views of the London and Madrid physicians. We think we are not biassed by national partiality, when we bestow our meed of approbation on the one, while we shudder at the *spasmodic* doctrines of the other.—*Ed.*

